# One new species of the *Clubiona obesa*-group from China, with the first description of *Clubiona kropfi* male (Araneae, Clubionidae)

**DOI:** 10.3897/zookeys.420.7770

**Published:** 2014-06-25

**Authors:** Pan-Long Wu, Feng Zhang

**Affiliations:** 1 The Key Laboratory of Invertebrate Systematics and Application, College of Life Sciences, Hebei University, Baoding, Hebei 071002, P. R. China

**Keywords:** Spiders, taxonomy, *Clubiona obesa*-group

## Abstract

The present paper describes two *Clubiona obesa*-group species: *Clubiona bicuspidata* **sp. n.** and the male *Clubiona kropfi* Zhang, Zhu & Song, 2003, which is described for the first time.

## Introduction

*Clubiona* Latreille, 1804, is the largest genus of the spider family Clubionidae. The genus encompasses approximately 465 species at present, is widely distributed around the world (except in South America) and has been revised both regionally and on a worldwide scale (Dondale and Redner 1976; Mikhailov 1990, 1991, 1995, 2002; Deeleman-Reinhold 2001; Platnick 2014). Because *Clubiona* is a large genus, several authors have suggested subdivisions of the genus into species groups (Simon 1932; Gertsch 1941; Lohmander 1944; Locket and Millidge 1953; Edwards 1958; Mikhailov 1995) and even subgenera (Lohmander 1944; Mikhailov 1990, 1991, 2002; Wunderlich 2011).

One of the largest species groups, *Clubiona obesa*, was first recognized by Edwards (1958) for the Nearctic species. This group is restricted to Asia and the Nearctic (Mikhailov 1995). Currently the group encompasses almost 50 species (Mikhailov 1995; Li and Wang 2014). Of these, 13 species occur in China (Li and Wang 2014): *Clubiona corrugata* Bösenberg & Strand, 1906, *Clubiona kurilensis* Bösenberg & Strand, 1906, *Clubiona lena* Bösenberg & Strand, 1906, *Clubiona manshanensis* Zhu & An, 1988, *Clubiona bakurovi* Mikhailov, 1990, *Clubiona kimyongkii* Paik, 1990, *Clubiona aciformis* Zhang & Hu, 1991, *Clubiona irinae* Mikhailov, 1991, *Clubiona fusoidea* Zhang, 1992, *Clubiona fuzhouensis* Gong, 1985, *Clubiona baishishan* Zhang, Zhu & Song, 2003, *Clubiona kropfi* Zhang, Zhu & Song, 2003 and *Clubiona lirata* Yang, Song and Zhu, 2003. This group is well studied in China, and only one species, *Clubiona kropfi*, is known by female sex. However, recently collected material has permitted us to recognize the previously unknown male of *Clubiona kropfi* Zhang et al., 2003 and to identify one species new to science. The goal of our paper is to provide a re-description of the *Clubiona kropfi* female and a first description of its male, and, additionally, describe a new species, *Clubiona bicuspidata* sp. n.

## Material and methods

All specimens were examined under a Tech XTL-II stereomicroscope. The drawings, photos and measurements were finished with a Leica M205A stereomicroscope equipped with a drawing tube and a DFC450 CCD camera. Carapace length was measured from the anterior margin to the posterior margin of the carapace medially. Eye sizes were measured as the maximum diameter of the lens in dorsal or frontal view. The measurements of legs are shown as total length (femur, patella, tibia, metatarsus, tarsus). The epigynum was cleared in a solution of potassium hydroxide (KOH) and transferred to 75% ethanol for drawing, taking photos and measuring. All measurements are in millimeters. All specimens studied are kept in 75% ethanol and deposited in the Museum of Hebei University (MHBU), Baoding, China.

The following abbreviations are used: ALE, anterior lateral eyes; AME, anterior median eyes; C, conductor; CO, copulatory openings; E, embolus; EP embolar part of bulbus; FD, fertilization ducts; MOA, median ocular area; PLE, posterior lateral eyes; PME, posterior median eyes; RTA, retrolateral tibial apophysis; S, spermathecae.

## Taxonomy

### Clubiona kropfi

Taxon classificationAnimaliaAraneaeClubionidae

Zhang, Zhu & Song, 2003

Figs 1 –12

Clubiona kropfi Zhang et al., 2003: 634, f. 2A–C (♀).

#### Type material.

Holotype ♀, China, Hebei Province, Laiyuan County, Baishi Mountain (39°12'N, 114°42'E), 16 July 1999, Feng Zhang leg., deposited in MHBU, examined.

#### Other material examined.

China: Hebei Province: Yu County, Xiaowutai Mountain (39°57'N, 114°48'E), 1 ♂ and 3 ♀, Shuigou Valley, 24 August 2012, Feng Zhang leg.; 1 ♂, Zhengjiagou Valley, 28 August 2012, Feng Zhang leg.; 1 ♂, Shuigou Valley, 5 July 2013, Panlong Wu leg.

#### Note.

This species was described on the basis of the holotype female with the male unknown.

#### Diagnosis.

This species is similar to *Clubiona bakurovi* (Mikhailov, 1990: f. 61–65), but can be distinguished by the hilt-like ventral branch of RTA, the tip of the embolus short and anti-clockwise, the EP wedge-shaped lacking a large tooth; the absence of epigynal grooves; the copulatory openings situated on the posterior edge of epigyne, and the septum thin.

#### Description.

Male. Total length 4.26–4.58. ♂ from Xiaowutai Mt: body 4.26 long; carapace 1.87 long, 1.31 wide; abdomen 2.24 long, 1.26 wide. Carapace yellowish. Head region slightly elevated above thorax. In dorsal view, anterior eye row slightly recurved, posterior eye row almost stright. Eye sizes and interdistances: AME 0.08, ALE 0.10, PME 0.08, PLE 0.10; AME–AME 0.04, AME–ALE 0.04, PME–PME 0.18, PME–PLE 0.12. MOA 0.27 long, front width 0.24, back width 0.35. Clypeus height 0.02. Chelicerae yellowish, promargin with six teeth, retromargin with three teeth. Endites yellow, longer than wide. Labium yellow brown, longer than wide. Abdomen oval, brown yellow, with conspicuous anterior tufts of hairs, dorsum with yellow thin hairs, cardiac pattern yellow brown; venter brown yellow. Spinnerets and legs yellow brown. Measurements of legs: leg I 4.60 (1.32, 0.65, 1.25, 0.86, 0.52), II 4.80 (1.40, 0.68, 1.33, 0.87, 0.52), III 4.23 (1.26, 0.56, 0.92, 1.02, 0.47), IV 6.17 (1.71, 0.67, 1.44, 1.77, 0.58). Male palp as in Figs 5–7, 10–12: RTA strongly expanded, forked, with hilt-like ventral branch; embolus arching behind tegulum and directing prolaterally; EP apophysis strong, wedge-shaped, with a triangular membrane proximally; conductor small, club-like, membranous.

**Figures 1–7.**
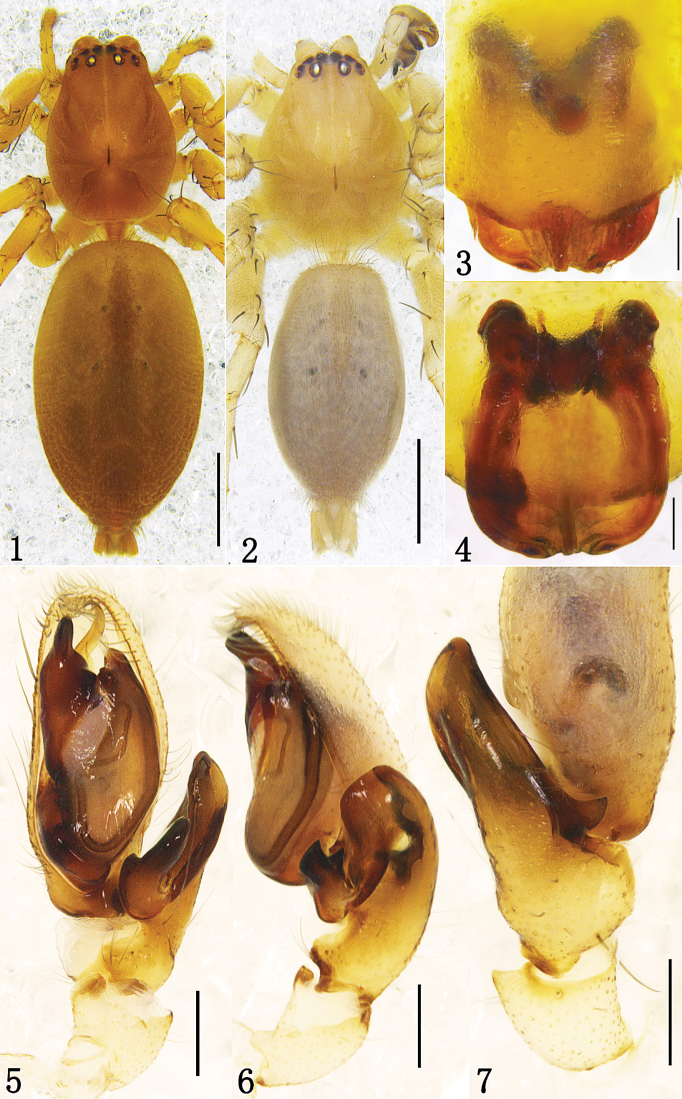
*Clubiona kropfi*, **1** female habitus, dorsal view **2** male habitus, dorsal view **3** epigyne, ventral view **4** vulva **5** left male palp, ventral view **6** same, retrolateral view **7** same, dorsal view, showing tibial apophysis. Scale bars: 1 mm (**1–2**); 0.1 mm (**3–4**); 0.2 mm (**5–7**).

**Figures 8–12.**
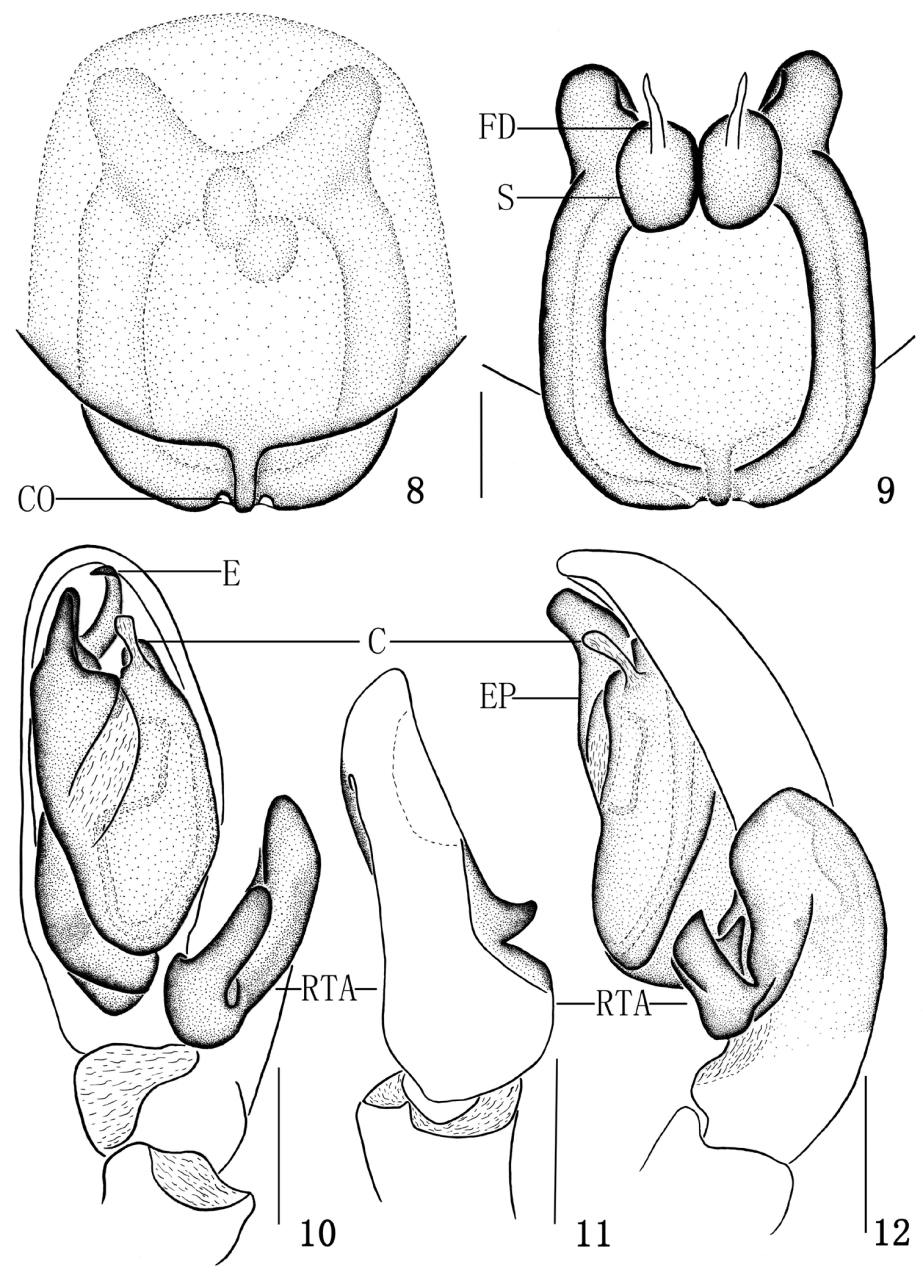
*Clubiona kropfi*, **8** epigyne, ventral view **9** vulva **10** left male palp, ventral view **11** tibial apophysis, dorsal view **12** left male palp, retrolateral view. Scale bars: 0.125 mm (**8–9**); 0.25 mm (**10–12**).

Female. Total length 4.73–4.98. ♀ from Xiaowutai Mt: body 4.98 long; carapace 1.82 long, 1.35 wide; abdomen 2.98 long, 1.79 wide. Eyes sizes and interdistances: AME 0.09, ALE 0.10, PME 0.08, PLE 0.10; AME–AME 0.05, AME–ALE 0.05, PME–PME 0.19, PME–PLE 0.13, ALE–PLE 0.07. MOA 0.28 long, front width 0.25, back width 0.41. Clypeus height 0.03. Labium 0.55 long, 0.24 wide. Endite 0.31 long, 0.24 wide. Measurements of legs: leg I 3.71 (1.10, 0.61, 0.90, 0.67, 0.43), II 3.90 (1.19, 0.63, 0.97, 0.68, 0.43), III 3.58 (1.09, 0.53, 0.72, 0.85, 0.39), IV 5.56 (1.60, 0.63, 1.24, 1.56, 0.53). Coloration darker than in male. Other characters as in male. Epigyne expanding posteriorly above epigastric groove, with a strongly sclerotized hind part. Copulatory openings separated from each other by a tongue-like process in the middle of the posterior part. Copulatory ducts directed laterad, then distad, almost parallel. Spermathecae spherical (Figs 3–4, 8–9).

#### Distribution.

China (Hebei).

### Clubiona bicuspidata sp. n.

Taxon classificationAnimaliaAraneaeClubionidae

http://zoobank.org/553FF41F-8AC9-4AE0-9B22-AB22D2A3E1DE

Figs 13 –19

#### Type material.

Holotype ♂, China: Xizang Autonomous Region (29°12'N, 94°12'E), Mainling County, Mingsheng Zhu leg., 18 August 2002 (collected in subadult stage, matured 29 August 2002). Paratype: 1 ♂, China: Shaanxi Province, Zhouzhi County, Taibai Mt (33°57'N, 107°45'E), 25 May 2009, Zhisheng Zhang leg.

#### Diagnosis.

The new species resembles *Clubiona baishishan* (Zhang et al., 2003: f. 1A–F), but differs by the shorter embolus, two pointed distal EP apophyses, and the tip of RTA without a concavity in dorsal view.

#### Etymology.

The species name is an adjective, derived from the shape of EP apophyses.

#### Description.

**Figures 13–16.**
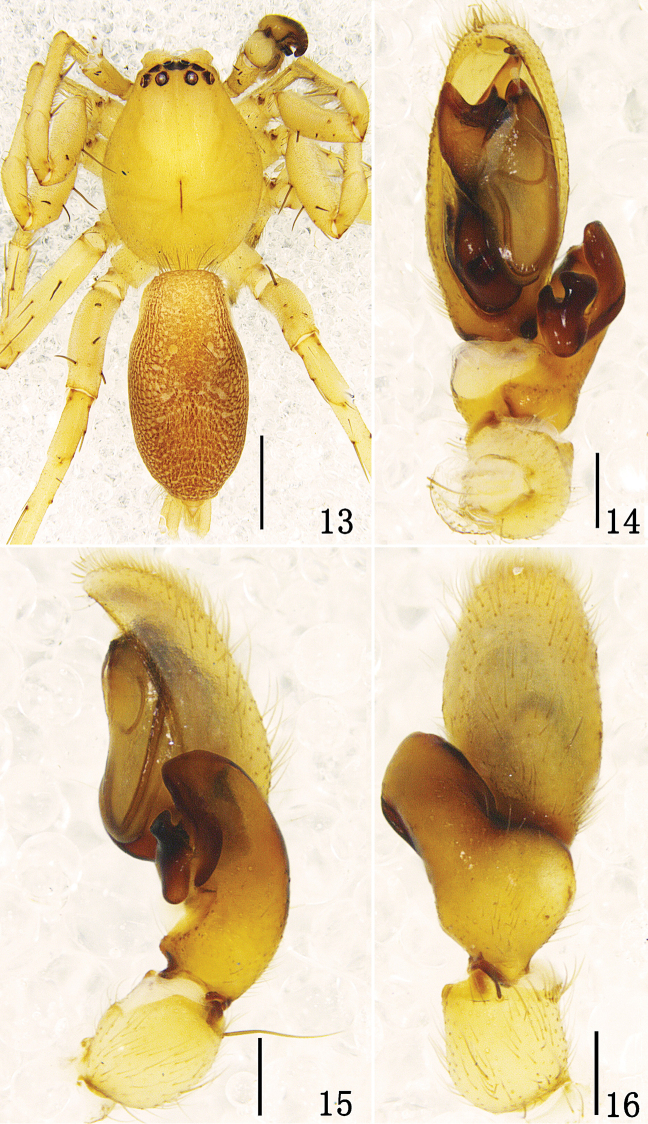
*Clubiona bicuspidata* sp. n. **13** male habitus, dorsal view **14** left male palp, ventral view **15** same, retrolateral view **16** same, dorsal view. Scale bars: 1 mm (**13**); 0.2 mm (**14–16**).

**Figures 17–19.**
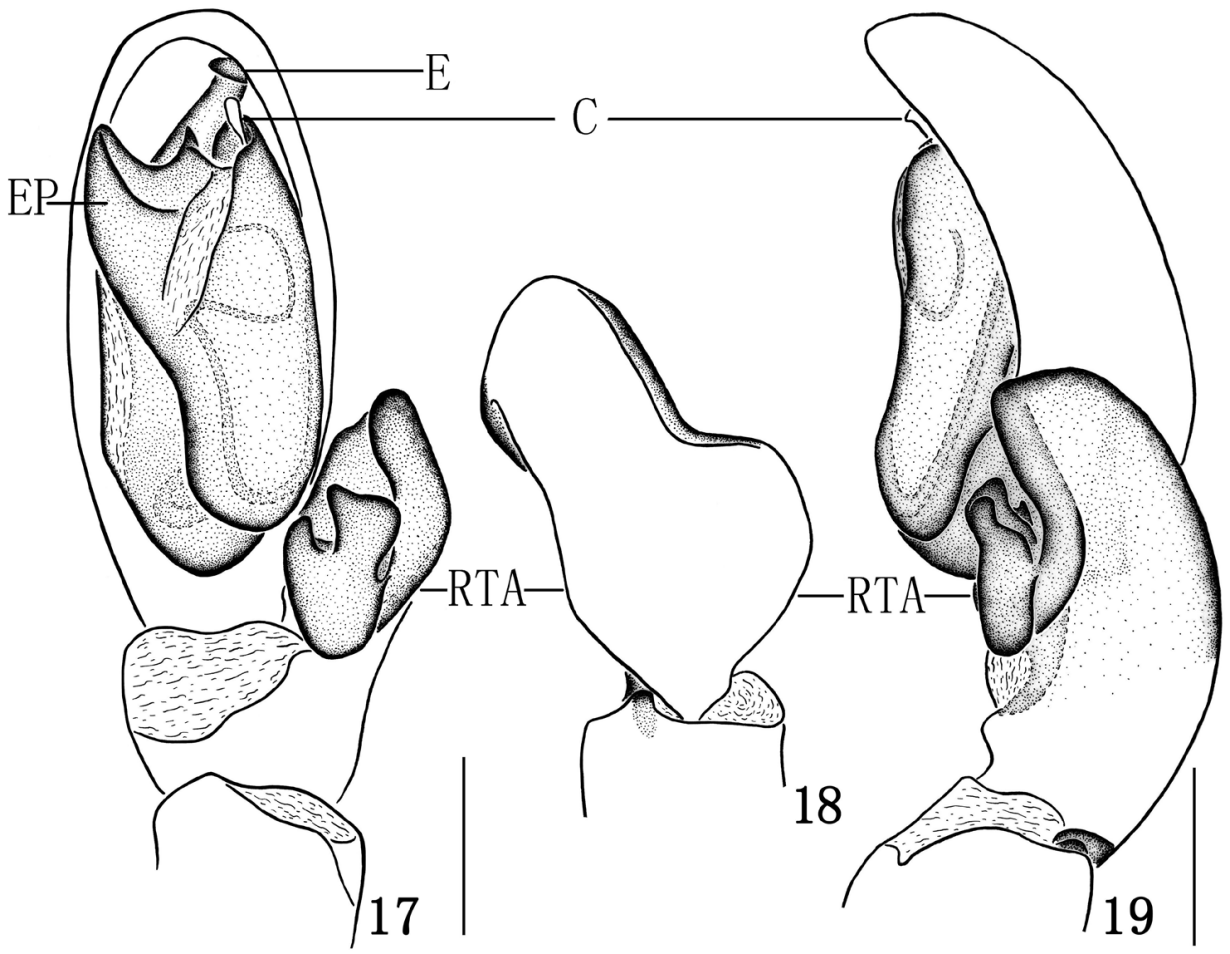
*Clubiona bicuspidata* sp. n. **17** left male palp, ventral view **18** tibial apophysis, dorsal view **19** left male palp, retrolateral view. Scale bars: 0.25 mm (**17–19**).

**Male.** Total length 4.65–4.74. Holotype: body 4.65 long; carapace 2.21 long, 1.69 wide; abdomen 2.43 long, 1.26 wide. Carapace (Fig. 13) yellow. Cephalic region yellowish, slightly elevated above thorax. Median furrow longitudinal. Anterior eye row slightly recurved (in dorsal view), posterior eye row almost straight. AME black, and other eyes white. Eyes sizes and interdistances: AME 0.12, ALE 0.13, PME 0.14, PLE 0.11; AME–AME 0.07, AME–ALE 0.04, PME–PME 0.22, PME–PLE 0.12, ALE–PLE 0.07. MOA 0.31 long, front width 0.28, back width 0.45. Clypeal height 0.03. Chelicerae yellow, promargin with five teeth, retromargin with three teeth. Endites yellowish, serrula dark. Labium yellow brown, 0.51 long, 0.28 wide. Abdomen tan, with white speckles; cardiac mark yellow brown. Spinnerets and legs yellow. Measurements of legs: leg I 5.83 (2.03, 0.86, 1.18, 1.10, 0.66), II 6.48 (2.05, 0.78, 1.85, 1.15, 0.65), III 5.12 (1.54, 0.57, 1.29, 1.28, 0.44), IV 7.35 (2.15, 0.60, 1.65, 2.28, 0.67). Male palp as in Figs 14–19: RTA strongly expanded, forked, ventral branch with two processes, one incus-shaped and the other thumb-shaped. Embolus arching behind tegulum and directing prolaterally. Tegulum with two pointed EP apophyses, one small and one large. Conductor small, rod-like, membranous.

**Female.** Unknown.

#### Distribution.

China (Xizang, Shaanxi).

## Supplementary Material

XML Treatment for Clubiona kropfi

XML Treatment for Clubiona bicuspidata
